# Dog Pulling on the Leash: Effects of Restraint by a Neck Collar vs. a Chest Harness

**DOI:** 10.3389/fvets.2021.735680

**Published:** 2021-09-06

**Authors:** Hao-Yu Shih, Clive J. C. Phillips, Daniel S. Mills, Yifei Yang, Fillipe Georgiou, Mandy B. A. Paterson

**Affiliations:** ^1^School of Veterinary Science, University of Queensland, Gatton, QLD, Australia; ^2^Curtin University Sustainability Policy Institute, Bentley, WA, Australia; ^3^School of Life Sciences, University of Lincoln, Lincoln, United Kingdom; ^4^School of Mathematical and Physical Sciences, University of Newcastle, Callaghan, NSW, Australia; ^5^Royal Society for the Prevention of Cruelty to Animals Queensland, Brisbane, QLD, Australia

**Keywords:** harness, collar, leash, tension, pull, dog, food, toy

## Abstract

Leash pulling is a concern for dog owners and can be detrimental to the health and welfare of dogs. Neck-collars and back-connection harnesses are popular restraint types. Harnesses have been proposed as a better and more considerate option for canine health and welfare. Anecdotally, dogs pull more when wearing a back-connection harness; however, there is no scientific evidence for this perception. This study aimed to investigate how strongly dogs pull on the lead to achieve a food treat or toy under restraint by a neck-collar versus a back-connection harness. A within-subject counterbalanced design was used for the study, involving 52 shelter dogs. A customised canine leash tension metre was connected to the collar or harness to record the pulling of the dogs, including measuring the maximal and mean leash tension, and the time spent pulling. In addition, dog behaviours were recorded using two cameras from two separate directions. The maximal and mean leash tension and the pulling time were greater under restraint by harness when attracting dogs with food treats. No significant difference between harness and collar was found in potential stress-related behaviours (e.g. tail and ear positions, lip-licking, and panting). However, dogs looked at the experimenter more often when restrained by harness than collar in the food treat attraction test. No significant difference was detected between harness and collar with respect to leash tension and stress-related behaviours in the toy attraction test. These findings suggest that dogs tend to pull stronger and more steadily when wearing a back-connection harness compared to a neck collar to reach the food treat but not the toy.

## Introduction

Compulsory leash policies, requiring dogs to be kept on-leash in public areas (1–3) have been implemented in many countries in order to protect wildlife (4), reduce disease transmission (5), and prevent dog attacks and involvement in traffic accidents (6, 7). Despite increasing emphasis on loose leash heelwork, many dogs still lunge and/or consistently pull on the leash during walks, especially when encountering stimuli of interest to them, such as food scraps or another dog (8). A survey with U.K. and Irish owners of pet dogs found that 82.7% of dogs pulled while on the lead (9). Pulling on the leash is also one of the most common problems reported in shelter dogs during the first month post-adoption (10).

Researchers have investigated the controlling effects and potential welfare concerns of different restraint types. For dogs wearing collars, excess pressure on the neck may cause musculoskeletal and tracheal injuries, and/or have negative effects on their eyes (11). Ogburn et al. (12) found that, compared to head collars, dogs were more disobedient on the leash while wearing traditional neck collars, although no significant differences in physiological responses, including blood pressure, pulse rate, respiratory rate, pupil diameter, and plasma cortisol concentrations, were detected. Nonetheless, dogs more frequently pawed at their noses and lowered their heads and ears when wearing the headcollar (12). Studies regarding the effect of harnesses on canine walking patterns are inconclusive. Lafuente et al. (13) found that harnesses influenced canine gait during walking and trotting by restricting their shoulder extension. Nagymáté et al. (14), however, reported that harnesses did not affect the dog's walking kinematics when they were walking off-leash or with a tense leash, and Grainger et al. (15) reported no difference in the gait and stress related signs when dogs walked or trotted in a harness vs. a plain neck collar.

There is a variety of equipment that owners use to walk their dogs, with flat-collars and back-connection harnesses being the most popular equipment choices (9). Harnesses may be a better restraint method, as the force exerted when wearing a harness is distributed over a larger area, while the force exerted on the neck when wearing a neck collar is more localised, increasing the potential for injury, or the exacerbation of existing medical conditions (16).

Despite plentiful literature concerning the potential animal welfare concerns stemming from different restraint methods, there is limited research of the effect of restraints on canine pulling behaviours. Anecdotally, back-connection harnesses are believed to relate to increased canine pulling on the leash [e.g., (17)]. This study examines this empirical hypothesis by investigating how strongly and for how long dogs pull on the leash to reach something they want while under restraint by a neck-collar vs. a back-connection harness. It was hypothesised that dogs would pull more strongly and for longer when the leash was connected to a harness compared to a neck collar, in line with common anecdotal opinion.

## Materials and Methods

This study was approved by the Animal Ethics Committee (Approval number: SVS/400/18) of The University of Queensland.

### Study Site

The research was conducted at the Royal Society for the Prevention of Cruelty to Animals, Queensland (RSPCA, QLD) shelter. Dogs were housed individually in rows of adjacent kennels (1.8 m wide × 1.2 m long × 3.0 m high) indoors and were able to make visual but not physical contact with one another across the central passage. Every enclosure was furnished with a metal crate, a raised mattress, a water bowl and enrichment articles (e.g., rubber toys or cardboard boxes). Each dog was walked twice daily, once in the morning between 08:00 and 10:00 h, and once in the afternoon between 14:00 and 16:00 h.

### Subjects

**Figure 1:**
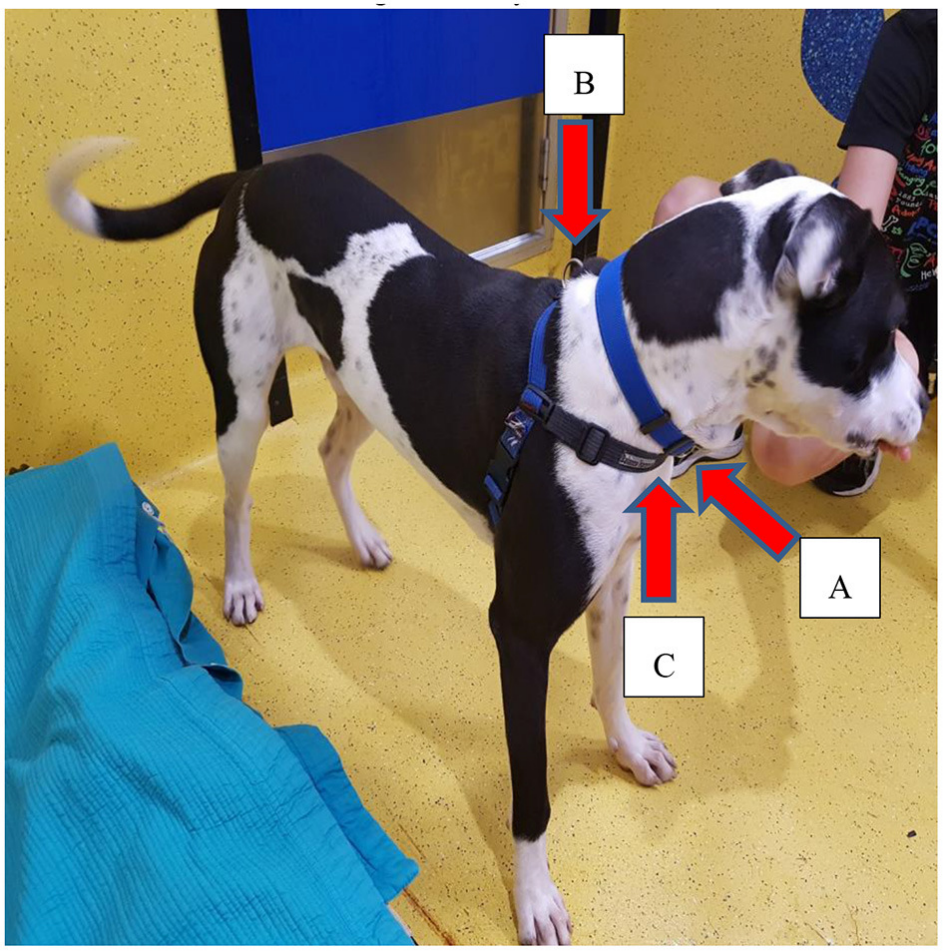
Dogs at RSPCA QLD wore both the collar and harness both during their daily walking routine and throughout the entire duration of the study. In this study, the leash tension metre (RobacScience, New South Wales, Australia) was connected to the collar **(A)** and harness **(B)** to test the pulling of dogs with both restraint types. The harness has one more front-attaching ring at the chest level **(C)** which is not shown in this picture.

This study involved 52 shelter dogs. All participating dogs had been resident at the RSPCA, QLD, for at least 1 week. The 52 subjects were 23 males and 29 females, ranging in age from 13 months to 11 years, and all dogs were gonadectomised. The body weight of the dogs ranged from 16 to 43 kg [median = 24 kg, interquartile range (IQR) = 7.76 kg]. All shelter dogs wore both the neck collar (plain neck collars of different brands) and the chest harness (Balance harness, Black Dog Wear Pty Ltd, Victoria, Australia) (Figure 1), and the leash was attached to both the collar and the harness in front of the chest of the dog (Figures 1A,C), during their daily walks (18), with a view to providing better control over the dog should they lunge (19). Therefore, all participant dogs were used to both restraint types and wore both restraints at all times during the study. Dogs with behavioural or medical issues (e.g., overtly aggressive or timid; suspected neck problems), as assessed by the RSPCA's behavioural modification team and veterinarians, that were deemed to be unsuitable for research were excluded from the study. Subjects were otherwise randomly selected from the adoption pens. Since each candidate dog lived individually in a pen with a pen number, a random number generator app (20) was used to randomly determine the participant dogs.

Each dog had an assigned walking level: levels 1, 2, 3, and 3+. These levels are assigned by RSPCA QLD staff based on their ease of walking on the leash during their daily walk. Level 1 dogs were those that walked on a loose leash most of the time. Level 2 was assigned to dogs pulling on the leash during the walk occasionally and displaying more undesirable behaviours than level 1 dogs. Level 3 was assigned to dogs which tended to pull on the leash fiercely and often, due to excitement or timidity. Level 3+ was reserved for dogs having severe behavioural issues, such as overt excitement or fearfulness, but which could still be managed by experienced volunteers; however, they were not assessed to necessarily pull on the leash with greater force than level 3 dogs. RSPCA QLD walking levels were ascribed as follows: one level 1, twenty-six level 2, twenty-one level 3, and four level 3+ dogs.

### Experimental Procedure

**Figure 2:**
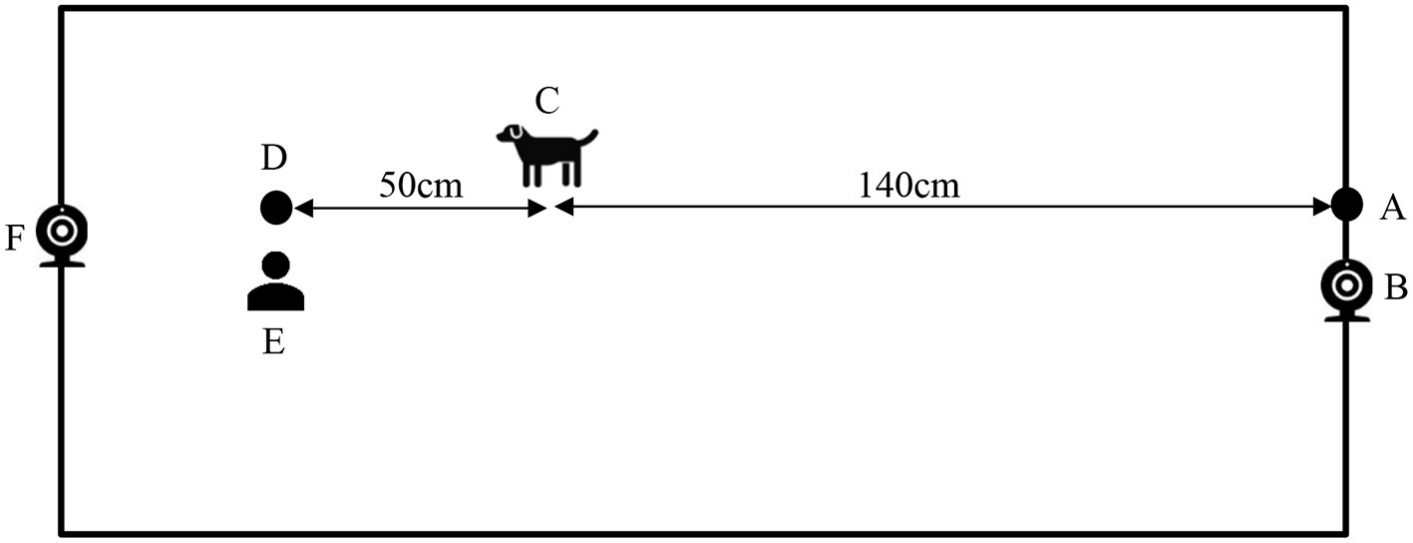
Illustration of the study area. The leash tension metre along with a l.4-metre-long dog leash was connected to the fixed point **(A)**, giving the dog an approximately 1.4-m maximal movement distance **(C)**. The attractant was placed on the floor 50 cm forward of the 1.4-metre maximal movement distance **(D)** and the experimenter knelt next to the attractant **(E)**. One camera was set next to the fixed point **(B)** and the other was set on the other side of the room **(F)**.

**Figure 3:**
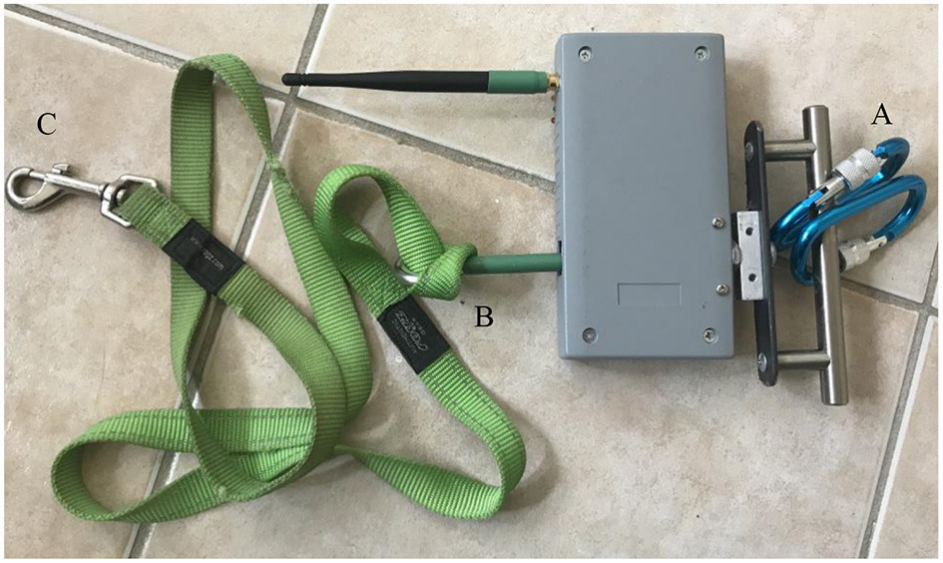
Demonstration of the leash tension metre. One end of the leash tension metre was connected to the tie-up ring using two carabiners **(A)**. On the other end of the device was a stainless-steel eyebolt to allow a simple connexion with a l.4-m-long commercial dog leash **(B)**, and the leash would be connected to the dog **(C)**.

A within-subject counterbalanced design was used in this study, with each individual dog acting as its own matched control. Participating dogs were taken to an air-conditioned experimental room away from distractions (e.g., other people and dogs), and were given 10 min to become accustomed to the environment. On one side of the room, at the starting point, a tie-up ring was secured on the wall (Figure 2A). The leash tension metre (RobacScience, New South Wales, Australia) (18) was connected to the tie-up ring using two carabiners (Anko, Australia) (Figure 3A). On the other end of the device was a stainless-steel eyebolt to allow a simple connection with a l.4-m-long commercial dog leash (Rogz Snake Lead) (Figure 3B) (18). This leash was then connected to the dog (Figure 3C), allowing the dog an ~1.4-metre maximal movement distance (Figure 2C). A camera (GoPro Hero 7 Silver, GoPro^®^, San Mateo, CA, USA) and an i-Phone 7 (Apple Inc., Cupertino, CA, USA) were set next to the tie-up ring on a cabinet (Figure 2B) and on the other side of the room (Figure 2F), respectively, to record the behaviours of the dog.

Before each trial, the leash was left unconnected to the dog for 10 s and the signals generated were used to calibrate the recorded data using MATLAB^®^ (MATLAB^®^ and Statistics Toolbox Release 2018b, The MathWorks, Inc., Natick, MA, USA). The experimenter then pulled the two ends of the tension metre and held the pull for 3 s by counting slowly “1, 2, 3.” This procedure was repeated three times in order to synchronise the tension data with the video. The leash was then connected to the collar (Figure 1A) or harness (Figure 1B) [randomly determined (21)] of the dog. The experimenter attracted the attention of the dog with a treat (Canine Care Australia, WA, Australia), slowly walking away and placing the treat on the ground 50 cm in front of the dog when the leash reached its full length (Figure 2D). Meanwhile, the experimenter knelt (Figure 2E) and faced away from the dog to avoid any cues that might influence the behaviour of the dog. Ten seconds after the treat was placed on the ground, the experimenter picked up the treat and guided the dog back to the starting point. The dog then took a 10 min break. After the break, the experimenter reconnected the leash to the neck collar or chest harness (whichever had not been connected the first time) and repeated the procedure again by attracting the dog with the treat. At the end of the food attraction test, the dog was rewarded with the treat and again given a 10 min break. After the second break, the experimenter reconnected the leash to either the neck collar or the chest harness [once more randomly determined (20)] and repeated the above two procedures, except this time attracting the dog with a toy; a squeaky tennis ball (KONG^®^, CO, USA). Four dogs were tested each day and the order of testing the dogs throughout the day was randomly determined (20).

### Behavioural Analysis

Two-hundred-and-six (food treat trials: *n* = 104; toy trials: *n* = 102) videos were coded in their entirety with Boris behaviour observation software (22) using a continuous recording method. One dog showed fear responses to the toy and thus the experimenter did not test it with the toy. All videos were coded by an independent observer who was unaware of the research hypothesis being tested. Coded behaviours were potential stress related signals, including tail and body positions, panting, and lip-licking as shown in Table 1.

**Table 1:** Ethogram of canine behaviour.

**Behaviour**	**Description**	**Behaviour type**
Ears position (23)	Relaxed—both ears of the dog hang relaxed. Upright—the dog holds its ears upright with tension. Flattened—the dog holds its ears flattened and back.	State event
Panting (24, 25)	The dog keeps its mouth wide open and breathes vigorously.	State event
Lip licking (24)	Part of tongue is shown and moved along the upper lip.	Point event
Looking at the experimenter (15, 26)	The dog turns head and looks towards the experimenter.	State event
Sniffing (26, 27)	The dog explores the ground, wall, or an object with its nose, or behaves it in the same way but as a stress-related signal.	State event
Shaking (28)	The dog shakes its body or head.	Point event
Paw lifting (24)	The dog raises one front limb.	State event
Tail position (26, 29, 30)	High—tail is held stiffly and upright, either curled over the back or straight. Neutral—tail is held in the normal carriage position for dog. Low—tail is held down either straight or slightly curled under the dogs' legs.	State event
Vocalisation (24, 31)	Dog vocalises, including barking, growling, whining and howling.	State event

*Parameters assessed: point event: the number of times the event was observed. State event: the duration of the observed event*.

### Leash Tension Analysis

Maximal and mean leash tension and the pulling time initiated by the dog were recorded by the leash tension metre. The pulling time was defined as the time dogs pulled on the leash with force greater than the threshold. Given the potential tissue damage to dogs when the leash tension was kept around 1% of body-weight-force, a threshold was set as 1% of body weight (32). Recorded data were processed using MATLAB^®^ (MATLAB^®^ and Statistics Toolbox Release 2018b, The MathWorks, Inc., Natick, MA, USA). For more details regarding the leash tension metre, please refer to Shih et al., (18).

### Statistical Analysis

Given the within-subject counterbalanced design, a Wilcoxon signed-rank test was initially used to evaluate our primary hypotheses concerning the difference between the harness and collar in terms of leash tension, pulling time, and behaviours expressed. This was followed by a multivariate analysis, to examine the main sources of variation contributing to the differences observed. Linear mixed-effects models followed by the backward elimination process were used for analyses. Where maximal and mean leash tension and the pulling time were considered as outcome variables, restraint types (collar/harness), the order of restraint type used, the order of each dog participating in the study during the day, and RSPCA canine walking level were entered into the models as predictors, and the ID of the dogs was set as a random effect. Normality of residual and the random effect were analysed using a normality plot and Shapiro–Wilk test and homogeneity of variance of residuals (VIF < 2). In line with the recommendations of Perneger, given the relative importance of type 1 vs. type 2 statistical errors on our exploration of potential factors of importance, no statistical correction for multiple testing was made (33).

## Results

### Leash Tension of Collar vs. Harness Conditions

Overall, the median maximal and mean leash tension were 0.62 kg-force/6.08 N (IQR = 2.56 kg-force/25.11 N) and 0.28 kg-force/2.75N (IQR = 0.39 kg-force/3.83N) respectively and the median proportion of pulling time was 0.23 (IQR = 0.86).

In the bivariate analyses, when testing dogs with food treats, the median maximal (*p* = 0.05) and mean (*p* = 0.0099) leash tension and the proportion of pulling time (*p* = 0.031) were significantly higher in the harness trial condition (maximal = 1.72 kg-force/16.87N; mean = 0.54 kg-force/5.3N; proportion of pulling time = 0.67) compared to the collar (maximal = 0.71 kg-force/6.97N; mean = 0.32 kg-force/3.14N; proportion of pulling time = 0.35) (Table 2). The proportion of pulling time is defined as the proportion of time dogs pulled on the leash with the force >1% of body weight. The threshold was set as 1% of body weight due to the potential tissue damage to dogs when the leash tension was kept around 1% of body-weight-force (32).

**Table 2:** Bivariate analysis (Wilcoxon signed-rank test) of leash tension between neck collar and chest harness conditions.

	**Food treat (*n* = 48)**	**Toy (*n* = 51)**
Max tension	Collar (IQR): 6.97 (4.71)N Harness (IQR): 16.87 (4.91)N ***p*****-value** **=** **0.05**	Collar (IQR): 3.14 (9.91)N Harness (IQR):3.63 (20.8)N *p*-value = 0.29
Mean tension	Collar (IQR): 3.14 (4.71)N Harness (IQR): 5.3 (4.91)N ***p*****-value** **=** **0.0099**	Collar (IQR): 1.18 (3.34)N Harness (IQR): 1.08 (4.02)N *p*-value = 0.46
Proportion of pulling time*	Collar (IQR): 0.35 (0.8) Harness (IQR): 0.67 (2) ***p*****-value** **=** **0.031**^+^	Collar (IQR): 0.085 (0.5) Harness (IQR): 0.02 (0.68) *p*-value = 0.65^+^

Some tension data was not recorded due to the loss of signal, leaving tension data of n = 48/52 dogs in the food treat tests and n = 51/52 dogs in the toy tests for analysis. *Proportion of time dogs pulled on the leash with the force greater than the threshold (1% of body weight). Threshold was set as 1% of body weight due to the potential tissue damage to dogs when the leash tension was kept around 1% of body-weight-force (32).

+ *Calculation was made using paired samples without ties. IQR, interquartile range. The median and interquartile range (IQR) of the maximal and mean leash tension, and the proportion of pulling time in the collar and harness trial conditions are presented*.

In the mixed-effect models, compared to the collar condition, the maximal (*p* = 0.0085) and mean (*p* = 0.0067) leash tension and the proportion of pulling time (*p*=0.028) were significantly higher in the harness condition during the food treat session (Table 3). In both food treat and toy sessions, the maximal (food treat: *p* = 0.019; toy: *p* = 0.0062) and mean (food treat: *p* = 0.022; toy: *p* = 0.017) leash tension and the proportion of pulling time (food treat: *p* = 0.011; toy: *p* = 0.017) significantly decreased in the second trial (Table 3).

**Table 3:** Linear mixed-effects model of the effects of restraint types and the order of restraint types used on leash tension and pulling time.

	**Max _**food**_**	**Mean _**food**_**	**Time _**food**_**	**Max _**toy**_**	**Mean _**toy**_**	**Time _**toy**_**
**Restraint type** (harness)	β: 0.38 SE:0.14 ***p*****: 0.0085**	β: 0.15 SE: 0.053 ***p*****: 0.0067**	β: 0.14 SE: 0.063 ***p*****: 0.028**	–	–	–
Order of restraint	β: −0.34 SE: 0.14 ***p*****: 0.019**	β: −0.12 SE: 0.053 ***p*****: 0.022**	β: −0.17 SE: 0.063 ***p*****: 0.011**	β: −0.15 SE: 0.054 ***p*****: 0.0062**	β: −0.13 SE: 0.053 ***p*****: 0.017**	β: −0.19 SE: 0.077 ***p*****: 0.017**
Order 1	Median: 12.85 N IQR: 42.58 N	Median: 4.51 N IQR: 4.71 N	Median: 0.7 IQR: 0.91	Median: 5 N IQR: 19.82 N	Median: 2.75 N IQR: 4.91 N	Median: 0.21 IQR: 0.83
Order 2	Median: 8.83 N IQR: 27.08 N	Median: 3.43 N IQR: 5.59 N	Median: 0.3 IQR: 0.93	Median: 1.67 N IQR: 8.04 N	Median: 0.91 N IQR: 2.35 N	Median: 0 IQR: 0.35

*Restraint type: collar was used for comparison. Order of restraint: the order of restraint types used was randomly determined. The RSPCA walking level and order of each dog participating in the study were also entered into the model but were excluded by the backward elimination process. Max _food:_ maximal leash tension during the food treat sessions (analysed after transformation to the power of 0.5). Mean _food:_ mean leash tension during the food treat session (analysed after transformation to the power of 0.5). Time _food:_ proportion of time that tension was greater than 1% of the bodyweight force within the 10 sec of testing period during the food treat session. Max _toy:_ maximal leash tension during the toy session (analysed after transformation to the power of 0.2). Mean _toy:_ mean leash tension during the toy session (analysed after transformation to the power of 0.5). Time _toy:_ proportion of time that tension was greater than 1% of the bodyweight force within the 10 sec of testing period during the toy session (analysed after transformation to the power of 0.5). β, regression coefficient. SE, standard error of β. p, p-value of the model. IQR, interquartile range. –, the predictor was excluded from the model due to the backward elimination process*.

### Behaviours Expressed in Collar vs. Harness Conditions

Paw-lifting, body shaking, sniffing, and vocalisation were seldom observed (Appendix Table 1); therefore these behaviours were excluded from analysis. Dogs looked at the experimenter significantly more frequently in the harness condition when tested with food treats (Wilcoxon signed-rank test: *p* = 0.011; mixed-effect model: *p* = 0.039) (Tables 4, 5).

**Table 4:** Bivariate analysis (Wilcoxon signed-rank test) of behaviours expressed in neck collar and chest harness conditions.

	**Food treat (*n* = 52)**	**Toy (*n* = 51)**
Lip-licking	Collar (IQR): 0.1 (0.2) Harness (IQR): 0.14 (0.29) *p*-value = 0.50^+^	Collar (IQR): 0.093 (0.2) Harness (IQR): 0.098 (0.2) *p*-value = 0.27^+^
Looking at experimenter	Collar (IQR): 0.11 (0.21) Harness (IQR): 0.16 (0.39) ***p*****-value** **=** **0.011**^**+**^	Collar (IQR): 0.14 (0.4) Harness (IQR): 0.15 (0.19) *p*-value = 0.96^+^
Panting	Collar (IQR): 0.73 (0.77) Harness (IQR): 0.72 (0.85) *p*-value = 0.2^+^	Collar (IQR): <0.01 (0.71) Harness (IQR): <0.01 (0.73) *p*-value = 0.91^+^
Ear-flatten	Collar (IQR): <0.01 (0.36) Harness (IQR): <0.01 (0.25) *p*-value = 0.63^+^	Collar (IQR): <0.01 (0.3) Harness (IQR): <0.01 (0.71) *p*-value = 0.5^+^
Tail-high	Collar (IQR): <0.01 (<0.01) Harness (IQR): <0.01 (<0.01) *p*-value = 0.52^+^	Collar (IQR): <0.01 (0.12) Harness (IQR): <0.01 (0.51) *p*-value = 0.79^+^

*Lip-licking: numbers of lip-licking observed per second. Looking at experimenter: proportion of time looking at the experimenter. Panting, proportion of time panting. Ear-flatten, proportion of time the dog kept its ears flatten. Tail-high: proportion of time the dog kept its tail in a high position*.

+ *Calculation was made using paired samples without ties. IQR, interquartile range. Paw-lifting, body shaking, sniffing, and vocalisation were seldomly observed*.

**Table 5:** Linear mixed-effects model of the effects of restraint types and the order of restraint types used on canine behaviours when testing with food treats.

**Food treat**					
	**Lip-licking**	**Looking at experimenter**	**Panting**	**Ear-flatten**	**Tail-high**
Restraint type (harness)	–	β: 0.087 SE: 0.041 ***p*****: 0.039**	–	–	–
Order of restraint	β: −0.073 SE: 0.031 ***p*****: 0.021**	–	–	–	–
Order 1	Median: 0.19 IQR: 0.19	Median: 0.15 IQR: 0.28	Median: 0.72 IQR: 0.79	Median: <0.01 IQR: 0.34	Median: <0.01 IQR: <0.01
Order 2	Median: 0.1 IQR: 0.2	Median: 0.11 IQR: 0.29	Median: 0.73 IQR: 1	Median: <0.01 IQR: 0.31	Median: <0.01 IQR: <0.01

*Restraint type: collar was used for comparison. Order of restraint: the order of restraint types used was randomly determined. The order of each dog participating in the study was also entered into the model but was excluded by the backward elimination process. Lip-licking: numbers of lip-licking observed per second (analysed after transformation to the power of 0.8). Looking at experimenter: proportion of time looking at the experimenter (analysed after transformation to the power of 0.5). Panting: proportion of time panting. Ear-flatten: proportion of time the dog kept its ears flatten. Tail-high: proportion of time the dog kept its tail in a high position. IQR, interquartile range. Paw-lifting, body shaking, sniffing, and vocalisation were seldomly observed. –: the predictor was excluded from the model due to the backward elimination process*.

*In the mixed-effect models, dogs displayed a significantly lower frequency of lip-licking behaviour in the second trial when tested with food treats (p = 0.021) and the toy (p=0.048) (Table 5 and Appendix Table 2). Finally, when testing with food treats, there was a significantly higher frequency of lip-licking behaviour during the first trial utilising the harness (p = 0.018) (Appendix Table 3) than during the second*.

## Discussion

### Leash Pulling

Our study supports the hypothesis that back-connection harnesses are associated with greater pulling on the leash. This finding is in line with the suggestion that dogs may be more comfortable when they wear a harness than a neck collar because the former enables the force exerted on the body to be more dispersed while the later causes localised pressure on the neck leading to increased discomfort (16).

When dogs were attracted with food treats, but not the toy, they created significantly higher leash tension and spent more time pulling when the leash was connected to the back-connection harness than to the neck collar. It may be that the toy was less appealing than food treats for many dogs, and/or dogs were generally less reactive to a toy when on-leash, since generally dogs do not play with a squeaky tennis ball when on-leash. It is also possible that the squeaky toy may be perceived as a foreign object that could potentially elicit more careful behaviour or even fear in shelter dogs as also seen in one of the participating dogs. Finally, there was an order effect since the toy test was done after the food treat test (12). It might be that dogs were habituated to the research process and thus were less responsive in the later toy tests.

### Behavioural Effects

In line with the previous study (15), we found no significant differences with respect to potential stress-related behaviours (e.g., lip-licking, panting, ears and tail position) between the collar and the harness. However, in the current study, dogs were tested in only a mildly to moderately excited state. Thus, we do not dismiss the concern with respect to the potential for tissue damage and health concerns resulting from the compression of the neck from a neck collar, especially when dogs are highly aroused (11, 34). Under the harness condition, dogs spent more time looking at the experimenter when tested with food treats and this might relate to differences in how the restraints are perceived. If the harness was more comfortable, their response may not be punished by the pulling, and so they may be less likely to stop the behaviour. Accordingly, they may be more likely to consider alternative solutions to the problem of accessing the resource, which may involve seeking assistance from humans through referential looking (35, 36). The other possible explanation may be that the localised force on the neck because of wearing a neck collar may restrict the head movement of dogs, causing them to be less likely to turn their heads to look at the experimenter. Also, dogs shift their weight depending on which side of their body the leash is on, and there may be a similar influence on the neck of the dog (37). However, in this study authors did not compare the effect of the harness vs. the neck collar and the effect of leash position on the range of motion and movement symmetry of the head or other body parts of the dog. Finally, given that a similar result was not observed when dogs were lured by the toy, and the force exerted by dogs was not as great, this might indicate that dogs did not value the toy as much as the food treat or could reflect carry-over effects from the first trial.

Dogs displayed fewer lip-licking behaviours in the second trial, again, possibly because they were more accustomed to the research procedure. Specifically, the frequency of lip-licking was higher during the first trial of the harness condition. It may be that the lip-licking in this context is not so much a sign of frustration (38), but actually a response to the appearance of the food treat and the lower deterrent effect of the harness; that is, an anticipatory response to the potential opportunity for food consumption (38). This would explain why other potential behavioural markers of distress did not increase as well.

The primary factor of interest was the role of restraint type (neck collar and back-connection harness) as a predictor of leash tension and behaviour regardless of the variation caused by other factors, which is why the data were analysed first using a simple bivariate analysis. Having established this, it is useful to evaluate the potential influence of other factors (39) such as the order of application of the restraint types used, the order of each dog's participation in the study during the day, and the dog's level of control when walking on-leash. Therefore, these factors were included in multivariable models for further analysis. This revealed that the order of use of the restraint type was significant and thus the importance of controlling for this in the current study and future investigations.

## Application and Future Study

Pressure sensors embedded in collars and harnesses have been utilised to assess the potential welfare and health impact of different restraint types on dogs. Such instruments measure the pressure experienced by the dog and the distribution of the pressure on the body of the dog (32, 40). However, the dog's experience may also be affected by that of the handler, who may jerk the leash, according to the tension they feel the dog is exerting (9, 41, 42). A leash tension metre, such as that used in this study, not only measures the tension of the leash but also differentiates the pulling direction, allowing us to quantify the pulling forces initiated by the dog and the handler respectively (18). Being pulled over by dogs is the most common form of dog-related musculoskeletal injury in persons in the U.K. (43), and frequent, long-term leash pulling may predispose handlers to upper limb injury such as shoulder pain, and hand sprain and strain (44). With a leash tension metre, it is possible to identify whether transient peak force or continuous pulling by a dog is more important to the experience of walking the dog and the risk of relevant injuries to either handler or dog. Another useful application of this device would be to explore the effects of other restraint types (e.g., different textures or widths of collar and different designs of collar and harness, especially those designed to reduce pulling) on canine leash pulling behaviour and owner response as a result.

In terms of our leash tension metre, the strain gauge (force sensor) and the accelerometer (direction sensor) used in this study were built into a box and held by the handler. Touching the leash with the other hand (the ideal way of walking dogs) would interfere with the measurement. Therefore, this device is designed for handlers to walk the dog using one hand only (18), and while walking dogs with a single hand is still very common for dog owners, it is not the most secure way to walk a dog. Alternatively, the sensors can be attached to the dog end of the leash, so that walking dogs with both hands to maximise control (especially with dogs who surge when on lead) would not affect the results. Dogs with this problem would be a useful population to study further. Nonetheless, even well-behaved dogs may occasionally surge towards a focus of interest and this study deliberately did not involve pulling initiated by the handler and is the first study to quantify the effect of restraint types on leash tension.

## Limitations

Limitations include the use of only shelter dogs; therefore, broad generalisations of the specific results should be made with caution. However, this population allowed us to work with a population that has experience of both forms of restraint, and is a strength of this study, since neither device on the dog was novel. However, we acknowledge that the history of restraint types used for each dog was unknown. Nonetheless these factors do not detract from the fact that this is the first study to quantify the leash pulling by dogs wearing collars and harnesses. Another limitation is that dogs seemed to be less responsive to the toy than the food treat, which was at the same time confounded by the testing order (dogs were always presented with food treat then toy). Future studies should consider randomising the testing object or confirm the interest of the dog to the object before testing. Finally, future studies might build on this by examining the effects of breed on the results, since some dog breeds have been specifically bred to pull objects (e.g., Alaskan malamutes and Siberian huskies), and may be more resistant to the pressure resulting from leash pulling (11).

## Conclusions

Dogs pulled harder and more steadily on the leash to reach food, but not toys when the leash was connected to the back-connection harness compared to the neck collar. Although increased pulling behaviour was related to the back-connection harness, this was not accompanied by a significant increase in potential stress-related responses.

## Data Availability Statement

The raw data supporting the conclusions of this article will be made available by the authors, without undue reservation.

## Ethics Statement

The animal study was reviewed and approved by Animal Ethics Committees (Approval number: SVS/400/18) of The University of Queensland.

## Author Contributions

H-YS, CP, DM, and MP: conceptualisation. H-YS, CP, DM, FG, and MP: methodology. FG, H-YS, and YY: software. H-YS: writing—original draft preparation and investigation. H-YS, YY, FG, and CP: validation, formal analysis, and data curation. MP and CP: resources. H-YS, DM, CP, MP, YY, and FG: writing—review and editing. H-YS and CP: visualisation and funding acquisition. MP, CP, and DM: supervision. H-YS and MP: project administration. All authors contributed to the article and approved the submitted version.

## Conflict of Interest

MP is employed as the principal scientist by RSPCA, Qld. The remaining authors declare that the research was conducted in the absence of any commercial or financial relationships that could be construed as a potential conflict of interest.

## Publisher's Note

All claims expressed in this article are solely those of the authors and do not necessarily represent those of their affiliated organizations, or those of the publisher, the editors and the reviewers. Any product that may be evaluated in this article, or claim that may be made by its manufacturer, is not guaranteed or endorsed by the publisher.
