# Supplementary material for: Dog Pulling on the Leash: Effects of Restraint by a Neck Collar vs. a Chest Harness
Source: Front Vet Sci. 2021 Sep 6;8:735680. doi: 10.3389/fvets.2021.735680 (PMC8450523; doi:10.3389/fvets.2021.735680)
Supplement: Supplementary file 1 [file Data_Sheet_1.docx]

Supplementary Material

# Appendix Table 1. Summary of rarely observed behaviours

| **Behaviour** | **Number of dogs observed expressing the behaviour in the food and toy trials respectively** |
| --- | --- |
| Paw-lifting | n = 5 (food); n = 2 (toy) |
| body shaking | n = 1 (food); n = 1 (toy) |
| sniffing | n = 10 (food); n = 12 (toy) |
| vocalization | n = 1 (food); n = 0 (toy) |

**Appendix Table 2. Linear mixed-effects model of the effects of restraint types and the order of restraint types used on canine behaviours when testing with toy.**

| **Toy** | | | | | |
| --- | --- | --- | --- | --- | --- |
|  | Lip-licking | Looking at  experimenter | Panting | Ear-flatten | Tail-high |
| **Restraint type**  (harness) | -- | -- | -- | -- | -- |
| **Order of**  **restraint** | *β*: -0.051  SE: 0.025)  ***p*: 0.048** | -- | -- | -- | -- |
| Order 1 | Median: 0.098  IQR: 0.2 | Median: 0.14  IQR: 0.21 | Median: <0.01  IQR: 0.74 | Median: <0.01  IQR: 0.71 | Median: <0.01  IQR: 0.79 |
| Order 2 | Median: <0.01  IQR: 0.19 | Median: 0.15  IQR: 0.44 | Median: <0.01  IQR: 0.6 | Median: <0.01  IQR: 0.3 | Median: <0.01  IQR: <0.01 |

Restraint type: collar was used for comparison. Order of restraint: the order of restraint types used was randomly determined. The order of each dog participating in the study was also entered into the model but was excluded by the backward elimination process. Lip-licking: numbers of lip-licking observed per second (analysed after transformation to the power of 0.8). Looking at experimenter: proportion of time looking at the experimenter. Panting: proportion of time panting (analysed after transformation to the power of 0.5). Ear-flatten: proportion of time the dog kept its ears flatten. Tail-high: proportion of time the dog kept its tail in a high position. IQR: interquartile range. Paw-lifting, body shaking, sniffing and vocalization were seldomly observed. --: the predictor was excluded from the model due to the backward elimination process.

**Appendix Table 3. Linear mixed-effects model of the effect of the interaction between restraint types and the order of restraint types used on canine behaviours when testing with food treats.**

| **Food treat** | | | | | |
| --- | --- | --- | --- | --- | --- |
|  | Lip-licking | Looking at  experimenter | Panting | Ears flatten | Tail-high |
| **Restraint type – order of restraint** |  | | | | |
| Collar-order 1 | Median: 0.14  IQR: 0.2  *β*: 0.044  SE: 0.044  *p*: 0.32 | Median: 0.13  IQR: 0.22  *β*: -0.074  SE: 0.059  *p*: 0.21 | Median: 0.96  IQR: 0.69  *β*: 0.073  SE: 0.074  *p*: 0.33 | Median: <0.01  IQR: 0.51  *β*: 0.0034  SE: 0.051  *p*: 0.95 | Median: <0.01  IQR: <0.01  *β*: -0.025  SE: 0.087  *p*: 0.77 |
| Harness-order 1 | Median: 0.2  IQR: 0.2  *β*: 0.12  SE: 0.05  ***p*: 0.018** | Median: 0.18  IQR: 0.28  *β*: -0.013  SE: 0.078  *p*: 0.87 | Median: 0.63  IQR: 0.76  *β*: -0.078  SE: 0.11  *p*: 0.49 | Median: <0.01  IQR: 0.22  *β*: -0.063  SE: 0.11  *p*: 0.59 | Median: <0.01  IQR: <0.01  *β*: -0.097  SE: 0.1  *p*: 0.35 |
| Collar-order 2 | Median: 0.1  IQR: 0.2  *β*: 0.016  SE: 0.05  *p*: 0.74 | Median: 0.091  IQR: 0.17  *β*: -0.11  SE: 0.078  *p*: 0.15 | Median: 0.63  IQR: 0.87  *β*: -0.049  SE: 0.11  *p*: 0.66 | Median: <0.01  IQR: 0.27  *β*: -0.059  SE: 0.12  *p*: 0.62 | Median: <0.01  IQR: <0.01  *β*: -0.18  SE: 0.11  *p*: 0.1 |
| Harness-order 2 | Median: 0.099  IQR: 0.2 | Median: 0.15  IQR: 0.43 | Median: 0.78  IQR: 0.96 | Median: <0.01  IQR: 0.37 | Median: <0.01  IQR: <0.01 |

Restraint type - order of restraint: the interaction of restraint types (collar/harness) and the order of restraint types used (order 1/ order 2). The order of each dog participating in the study was also entered into the model but was excluded by the backward elimination process. Lip-licking: numbers of lip-licking observed per second (analysed after transformation to the power of 0.8). Looking at experimenter: proportion of time looking at the experimenter (analysed after transformation to the power of 0.5). Panting: proportion of time panting. Ear-flatten: proportion of time the dog kept its ears flatten. Tail-high: proportion of time the dog kept its tail in a high position. IQR: interquartile range. Paw-lifting, body shaking, sniffing and vocalization were seldomly observed. --: the predictor was excluded from the model due to the backward elimination process.

**Appendix Table 4. Linear mixed-effects model of the effect of the interaction between restraint types and the order of restraint types used on the leash tension and the proportion of pulling time.**

|  | Max _food_ | Mean _food_ | Time _food_ | Max _toy_ | Mean _toy_ | Time _toy_ |  |
| --- | --- | --- | --- | --- | --- | --- | --- |
| **Restraint type – order of restraint** |  | | | | | | |
| Collar-order 1 | Median: 1.06 kg-force/ 10.4N  IQR: 2.72 kg-force/ 26.68N  *β*: -0.043  SE: 0.19  *p*: 0.82 | Median: 0.38 kg-force/ 3.73N  IQR: 0.49 kg-force/ 4.81N  *β*: -0.024  SE: 0.073  *p*: 0.74 | Median: 0.48 kg-force/ 4.71N  IQR: 0.85 kg-force/ 8.34N  *β*: 0.023  SE: 0.089  *p*: 0.79 | Median: 0.65 kg-force/ 6.38N  IQR: 2.3 kg-force/ 22.56N  *β*: 0.11  SE: 0.083  *p*: 0.2 | Median: 0.35 kg-force/ 3.43N  IQR: 0.48 kg-force/ 4.71N  *β*: 0.11  SE: 0.082  *p*: 0.2 | Median: 0.24 kg-force/ 2.35N  IQR: 0.85 kg-force/ 8.34N*β*: 0.21  SE: 0.11  *p*: 0.064 |  |
| Harness-order 1 | Median: 2.17 kg-force/ 21.29N  IQR: 5.06 kg-force/ 49.64N  *β*: 0.22  SE: 0.27  *p*: 0.41 | Median: 0.55 kg-force/ 5.4N  IQR: 0.4 kg-force/ 3.92N  *β*: 0.086  SE: 0.097  *p*: 0.38 | Median: 0.73 kg-force/ 7.16N  IQR: 0.69 kg-force/ 6.77N  *β*: 0.15  SE: 0.11  *p*: 0.18 | Median: 0.46 kg-force/ 4.51N  IQR: 2 kg-force/ 19.62N  *β*: -0.0078  SE: 0.1  *p*: 0.94 | Median: 0.19 kg-force/ 1.86N  IQR: 0.45 kg-force/ 4.41N  *β*: 0.0098  SE: 0.089  *p*: 0.91 | Median: 0.12 kg-force/ 1.18N  IQR: 0.78 kg-force/ 7.65N  *β*: 0.13  SE: 0.11  *p*: 0.24 |  |
| Collar-order 2 | Median: 0.61 kg-force/ 5.98N  IQR: 0.89 kg-force/ 8.73  *β*: -0.49  SE: 0.27  *p*: 0.066 | Median: 0.16 kg-force/ 1.57N  IQR: 0.45 kg-force/ 4.41N  *β*: -0.19  SE: 0.097  *p*: 0.057 | Median: 0.095 kg-force/ 0.93N  IQR: 0.66 kg-force/ 6.47N  *β*: -0.16  SE: 0.11  *p*: 0.17 | Median: 0.2 kg-force/ 1.96N  IQR: 0.59 kg-force/ 5.79  *β*: -0.2  SE: 0.1  *p*: 0.051 | Median: 0.093 kg-force/ 0.91N  IQR: 0.18 kg-force/ 1.77N  *β*: -0.14  SE: 0.089  *p*: 0.12 | Median: 0 kg-force/ 0N  IQR: 0.24 kg-force/ 2.35N  *β*: -0.042  SE: 0.11  *p*: 0.71 |  |
| Harness-order 2 | Median: 1.51 kg-force/ 14.81N  IQR: 4.44 kg-force/ 43.56N | Median: 0.5 kg-force/ 4.91N  IQR: 0.58 kg-force/ 5.69N | Median: 0.43 kg-force/ 4.22N  IQR: 1 kg-force/ 9.81N | Median: 0.16 kg-force/ 1.57N  IQR: 2.46 kg-force/ 24.13N | Median: 0.094 kg-force/ 0.92N  IQR: 0.37 kg-force/ 3.63N | Median: 0 kg-force/ 0N  IQR: 0.38 kg-force/ 3.73N |  |

Restraint type - order of restraint: the interaction of restraint types (collar/harness) and the order of restraint types used (order 1/ order 2). The order of each dog participating in the study and the RSPCA walking level were also entered into the model but were excluded by the backward elimination process. Max _food:_ maximal leash tension during the food sessions (analysed after transformation to the power of 0.5). Mean _food:_ mean leash tension during the food session (analysed after transformation to the power of 0.5). Time _food:_ Proportion of time that tension was greater than 1% of the bodyweight force within the 10 sec of testing period during the food session. Max _toy:_ maximal leash tension during the toy session (analysed after transformation to the power of 0.2). Mean _toy:_ mean leash tension during the toy session (analysed after transformation to the power of 0.5). Time _toy:_ Proportion of time that tension was greater than 1% of the bodyweight force within the 10 sec of testing period during the toy session (analysed after transformation to the power of 0.5). *β*: regression coefficient. SE: standard error of *β*. *p*: p-value of the model. IQR: interquartile range.

**Appendix Table 5. Linear mixed-effects model of the effect of the interaction between restraint types and the order of restraint types used on canine behaviours when testing with toy.**

| **Toy** | | | | | |
| --- | --- | --- | --- | --- | --- |
|  | Lip-licking | Looking at  experimenter | Panting | Ears flatten | Tail-high |
| **Restraint type – order of restraint** |  | | | | |
| Collar-order 1 | Median: 0.098  IQR: 0.2  *β*: 0.022  SE: 0.038  *p*: 0.56 | Median: 0.12  IQR: 0.22  *β*: -0.062  SE: 0.063  *p*: 0.34 | Median: 0.033  IQR: 0.89  *β*: 0.083  SE: 0.071  *p*: 0.25 | Median: <0.01  IQR: 0.17  *β*: 0.04  SE: 0.076  *p*: 0.6 | Median: <0.01  IQR: 0.84  *β*: 0.074  SE: 0.078  *p*: 0.35 |
| Harness-order 1 | Median: 0.1  IQR: 0.2  *β*: 0.047  SE: 0.044  *p*: 0.29 | Median: 0.16  IQR: 0.17  *β*: -0.0063  SE: 0.079  *p*: 0.94 | Median: <0.01  IQR: 0.73  *β*: 0.012  SE: 0.12  *p*: 0.92 | Median: 0.062  IQR: 0.83  *β*: 0.13  SE: 0.13  *p*: 0.31 | Median: <0.01  IQR: <0.01  *β*: 0.03  SE: 0.12  *p*: 0.81 |
| Collar-order 2 | Median: <0.01  IQR: 0.19  *β*: -0.026  SE: 0.044  *p*: 0.56 | Median: 0.17  IQR: 0.44  *β*: 0.031  SE: 0.079  *p*: 0.69 | Median: <0.01  IQR: 0.59  *β*: -0.012  SE: 0.12)  *p*: 0.92 | Median: <0.01  IQR: 0.27  *β*: 0.015  SE: 0.13  *p*: 0.91 | Median: <0.01  IQR: <0.01  *β*: -0.09  SE: 0.13  *p*: 0.48 |
| Harness-order 2 | Median: 0.049  IQR: 0.19 | Median: 0.14  IQR: 0.32 | Median: <0.01  IQR: 0.73 | Median: <0.01  IQR: 0.22 | Median: <0.01  IQR: 0.62 |

Restraint type - order of restraint: the interaction of restraint types (collar/harness) and the order of restraint types used (order 1/ order 2). The order of each dog participating in the study was also entered into the model but was excluded by the backward elimination process. Lip-licking: numbers of lip-licking observed per second (analysed after transformation to the power of 0.8). Looking at experimenter: proportion of time looking at the experimenter (analysed after transformation to the power of 0.5). Panting: proportion of time panting. Ear-flatten: proportion of time the dog kept its ears flatten. Tail-high: proportion of time the dog kept its tail in a high position. IQR: interquartile range. Paw-lifting, body shaking, sniffing and vocalization were seldomly observed. --: the predictor was excluded from the model due to the backward elimination process.
